# Dynamic regulation of neutrophil immunometabolism by platelet-derived metabolites

**DOI:** 10.3389/fimmu.2025.1542438

**Published:** 2025-03-27

**Authors:** Manuel Alejandro Mosso-Pani, Dante Barreda, Ma. Isabel Salazar

**Affiliations:** ^1^ Departamento de Microbiología, Escuela Nacional de Ciencias Biológicas, Instituto Politécnico Nacional, Ciudad de Mexico, Mexico; ^2^ Laboratory of Genomics, ODIN Bioscience, Miami, FL, United States; ^3^ Aging and Metabolism Research Program, The Oklahoma Medical Research Foundation, Oklahoma City, OK, United States; ^4^ Laboratorio Nacional de Vacunología y Virus Tropicales (LNVyVT), Escuela Nacional de Ciencias Biológicas Instituto Politécnico Nacional, Ciudad de México, Mexico

**Keywords:** neutrophil immunometabolism, platelet-derived metabolites, mitochondrial metabolism, platelet-neutrophil aggregates, inflammation regulation

## Abstract

Platelets, traditionally known for their roles in hemostasis and thrombosis, have emerged as key regulators of immune responses, particularly through their dynamic interactions with neutrophils. This review explores how platelets influence neutrophil functions by forming platelet-neutrophil aggregates, releasing extracellular vesicles, and secreting metabolites. These processes govern critical immune activities, including cell recruitment, activation, endothelium interactions and the resolution or exacerbation of inflammation. Additionally, platelets induce metabolic reprogramming in neutrophils, affecting glycolysis and mitochondrial pathways, while also shaping the immune microenvironment by modulating other immune cells, such as T and B cells. Understanding this complex crosstalk between platelets and neutrophils—two of the most abundant cell types in the bloodstream—might reveal new therapeutic opportunities to regulate immune responses in inflammatory and immune-mediated diseases.

## 1 Introduction

Platelets, derived from megakaryocytes in the bone marrow, are released into the bloodstream (1). Despite lacking nuclei, they play critical roles in hemostasis, maintaining endothelial integrity (2), and immunity. Beyond their ‘so called’ classical functions, growing experimental evidence identifies platelets as key contributors to immunity, highlighting their ability to modulate both acute and chronic inflammation (3, 4).

Neutrophils, the most abundant innate immune cells, are rapidly mobilized to sites of injury, guided by chemokine gradients secreted by different cellular types, including damaged endothelial cells (5), tissue macrophages, and platelets (6). Platelets bound to neutrophils enhance their mobilization to these sites. Once at the inflammation site, activated neutrophils contribute to pathogen clearance through specialized mechanisms such as phagocytosis, cytokine release, oxidative burst, and the secretion of neutrophil extracellular traps (NETs) (5). The emerging field of immunometabolism examines how cellular metabolism shapes immune responses. The basics of immunometabolism suggests that pro-inflammatory responses are typically driven by glycolysis, whereas anti-inflammatory responses are primarily fueled by oxidative phosphorylation (OxPhos) (7, 8). For example, accumulation of Krebs cycle intermediates, such as succinate and citrate, along with mitochondrial reactive oxygen species (ROS), trigger the activation of the NLRP3 inflammasome which in turn processes interleukin-1β (IL-1β), promoting a pro-inflammatory state. In contrast, IL-10 activates mitochondrial metabolism via AMP-activated protein kinase (AMPK), which stimulates both fatty acid oxidation (FAO) and OxPhos. This metabolic shift leads to the consumption of Krebs cycle intermediates and a reduction in mitochondrial ROS production, inhibiting NLRP3 inflammasome assembly, and preventing IL-1β processing, thereby fostering an anti-inflammatory response (9). This observation has raised the idea that repolarizing immune cells towards a non-inflammatory phenotype by modulating cellular metabolism using metabolic intermediated molecules is possible.

Although neutrophil metabolism has traditionally been viewed as largely dependent on glycolysis, recent evidence suggests that mitochondrial metabolism also plays a role in regulating their effector functions (10). Furthermore, the impact of these metabolic pathways on inflammatory and anti-inflammatory responses remains an important area for further investigation. Emerging evidence suggests that platelets contribute to this metabolic reprogramming of neutrophils, enhancing their immune responses due to their close anatomical proximity through two primary mechanisms: direct interactions via receptor-ligand binding and indirect interactions through the release of soluble molecules. These platelet-neutrophil interactions are essential for the metabolic and functional modulation of neutrophils, amplifying their immune activity (11, 12).

This review highlights the current understanding of the crosstalk between platelets and neutrophils, focusing on how platelets regulate neutrophil immune responses. Additionally, we discuss the role of platelet-derived metabolites in driving the metabolic reprogramming of neutrophils and how this reprogramming impacts their effector functions.

## 2 Glycolysis and the pentose phosphate pathway in neutrophil immunometabolism

Glycolysis is the predominant metabolic pathway during neutrophil immune responses, given the short lifespan of neutrophils (13, 14). Glycolysis begins when neutrophils, take up glucose via GLUT transporters, primarily GLUT1 and GLUT3, and convert it into pyruvate through a series of enzymatic reactions. The conversion yields two molecules of ATP, one molecule of NADH, and intermediates for the pentose phosphate pathway (PPP). In following metabolic reactions, the resulting products can proceed under two different conditions depending on oxygen availability. Under aerobic conditions, pyruvate is oxidized to acetyl coenzyme A (acetyl-CoA) through the Krebs cycle, producing 32 molecules of ATP along with other reduced intermediates. Under hypoxic conditions, pyruvate is reduced to lactate by lactate dehydrogenase, a process that not only sustains glycolysis but also serves as a metabolic foundation for immune cells to produce and secrete soluble molecules. These molecules actively contribute to establishing a pro-inflammatory microenvironment, linking metabolic adaptation to immune function.

Neutrophils harness glycolysis to support their effector functions. Upon activation by stimuli such as phorbol 12-myristate 13-acetate (PMA), neutrophils increase glucose uptake in proportion to the surface expression of GLUT-1. This metabolic shift triggers critical processes, including the production and release of NETs (15). NET formation is a highly regulated process that involves chromatin decondensation, histone citrullination, and the preparation of nuclear and mitochondrial DNA for expulsion (16, 17). These web-like structures play a pivotal role in trapping and neutralizing pathogens. Additionally, the metabolic activity in neutrophils provides the energy and intermediates required for such immune processes, highlighting the integral connection between cellular metabolism and the ability of neutrophils to combat infections.

Although the abrogation of neutrophil effector functions by glucose depletion has been reported (15, 18), under conditions of inflammation or hypoxia, neutrophils rely on intracellular glycogen stores accumulated through gluconeogenesis (12). These glycogen stores are essential for neutrophil survival and efficient microbial killing via the production and release of NETs. Impaired glycogenolysis is associated with reduced ability of neutrophils to produce NETs (12, 19). In COVID-19 patients, for example, increased glycogenolysis is linked to the release of large amounts of NETs into the bloodstream, contributing to the exacerbation of the inflammatory state (19).

Similar to glycolysis, the PPP operates in the cytosol and plays a crucial role in neutrophil metabolism by supporting both biosynthetic and antimicrobial functions. It produces ribose-5-phosphate for DNA and RNA synthesis, glycerol-3-phosphate for phospholipid synthesis, and NADPH molecules essential for neutrophil oxidative burst and antimicrobial activity. Glucose-6-phosphate dehydrogenase (G6PDH), the key enzyme in the PPP, facilitates the conversion of glucose-6-phosphate to ribulose-5-phosphate while generating two molecules of NADPH. This NADPH powers NADPH oxidases (NOX2), which catalyze the reduction of molecular oxygen to superoxide, leading to the production of ROS. These ROS are crucial for the oxidative burst that enables pathogen clearance during phagocytosis (12).

A shift toward increased PPP activity ensures sufficient NADPH supply for ROS production while supporting neutrophil survival and function (20, 21). This includes *de novo* protein synthesis, cytokine secretion, and sustained antimicrobial capacity in inflammatory microenvironments (20).

## 3 Mitochondrial regulation of neutrophil immunometabolism

Emerging experimental evidence in neutrophil immunometabolism suggests that mitochondrial metabolism is also active in these cells and plays crucial roles in development, survival, and effector functions (22), despite the relatively low abundance of mitochondria in neutrophils (23). Mitochondrial metabolism encompasses OxPhos, wherein electron transport chain (ETC) complexes (complex I to IV) couple with ATP synthesis, generating mitochondrial transmembrane potential and relying on metabolic intermediates supplied by the Krebs cycle. Disruption of mitochondrial function impairs energy metabolism, significantly affecting not only the ontogeny (24) and differentiation (25) of neutrophils but also their immune functions. Immature neutrophils, which exhibit lower glucose uptake, rely on mitochondrial metabolism for ROS generation (26). Moreover, inhibition of ETC complex III significantly reduces antimicrobial activity (27) and oxygen consumption, correlating with a marked decrease in ROS production (28, 29).

Furthermore, mitochondria house cytotoxic proteins and monitor metabolic and redox states, accumulating and storing Ca^2+^ to facilitate apoptotic processes without triggering release of toxic contents into the extracellular milieu (21). These mitochondrial processes promote neutrophil polarization, chemotaxis (30), and rely on maintenance of mitochondrial membrane potential and ATP synthase function. Consequently, inhibition of mitochondrial activity hampers neutrophil transmigration to tissues (31).

If mitochondrial dysfunction occurs, a premature apoptosis process is activated in neutrophils. Conversely, increased mitochondrial activity has been linked to heightened mitochondrial ROS production during colitis (32). Thus, tight regulation of neutrophil mitochondrial function is pivotal to maintaining homeostasis. There is experimental evidence that mitochondrial morphology/physiology can be molded by platelet factors (30, 33). In the future it would be interesting to explore how to manipulate an immune response mediated by neutrophils by regulating their mitochondrial function using platelets. When platelets are absent, neutrophils exhibit increased surveillance, which may contribute to the development of chronic inflammation and fibrosis (34).

### 3.1 Oxidative burst

One key mechanism of neutrophils to eliminate infectious agents is the activation of the oxidative burst, a process tightly regulated by mitochondrial activity. Upon stimulation, NADPH oxidase assembles at the cell membrane and becomes activated, catalyzing the NADPH-dependent reduction of O_2_ to form superoxide anions (O2−) and derivatives such as H_2_O_2_, hydroxyl radicals (OH•), and hypochlorous acid (HOCl) (35). Mitochondrial ROS production is induced by N-formyl peptides (fMLP), which are exclusively expressed on bacteria (36). Notably, inhibiting mitochondrial function reduces neutrophil oxidative burst and ROS production in response to fMLP (37). Additionally, mitochondria-targeted antioxidants such as SkQ1 can also inhibit fMLP-induced neutrophil oxidative burst and degranulation (36).

NADPH production is not limited to the PPP. In neutrophils, alternative pathways and enzymes also contribute to meeting the high demand for ROS generation (38, 39). For instance, the malic enzyme catalyzes the conversion of malate, a Krebs cycle intermediate, into NADPH (40). Additionally, isocitrate dehydrogenases 1 and 2, as well as pathways such as the folate cycle, may enhance NADPH availability, further supporting ROS production (41). In conditions like acute destructive pancreatitis, both the PPP and the Krebs cycle are significantly upregulated in neutrophils, highlighting the critical role of these metabolic pathways in sustaining the oxidative burst.

Mitochondria also contribute to ROS production by linking NADPH oxidase (NOX2) activity to metabolic processes (26, 27, 42). This connection relies on an ETC Complex III-dependent mechanism (27), coupled with increased FAO (26). During FAO, free fatty acids are metabolized to generate acetyl-CoA, which fuels the Krebs cycle and sustains NADPH production (26). Notably, neutrophils adapt to metabolic stress—such as hyperglycemia or limited glucose and glutamine availability—by relying on mitochondrial FAO to maintain ROS generation (26, 40, 43) and NETs production (44). This metabolic flexibility highlights the critical interplay between mitochondrial and cytosolic pathways in regulating neutrophil bactericidal activity under various physiological and pathological conditions.

### 3.2 Neutrophil extracellular traps production

The activity of the ETC complexes I and III drives the production and secretion of NETs neutrophil activation by platelet-activated factor (PAF) (45) intrinsically or by lipopolysaccharide (LPS) through a Toll-like receptor 4 (TLR-4) dependent manner during infection (27).

PAF, a key pro-inflammatory mediator, enhances neutrophil activation and ROS production by mitochondria, modulating Ca^2+^ signaling (46, 47). NETs production and secretion are also increased by high mobility group box 1 (HMGB1), a key regulator of cell bioenergetics that modulates the balance between apoptosis and autophagy (48). In neutrophils, HMGB1 regulates recruitment, activation, and survival by preventing mitochondrial potential reduction and inducing autophagosome formation (49). Blocking autophagic flux prevents HMGB1-induced NETs production and secretion (49). Furthermore, in obese mice, NETs formation shifts from a sole reliance on glycolysis to ATP production through mitochondrial FAO and the PPP (50, 51). This process also depends on the catabolism of glutamate and proline (52, 53), underscoring the metabolic adaptability of neutrophils in supporting NETs formation.

Our central hypothesis is that mitochondrial function plays a critical role in regulating neutrophil activity and its dysregulation has significant pathological implications. When mitochondrial dysfunction occurs, it triggers premature apoptosis in neutrophils, impairing their immune response. In contrast, increased mitochondrial activity has been associated with elevated mitochondrial ROS production, as observed in conditions such as colitis (32). External factors, including platelet-mediated regulation, may modulate neutrophil mitochondrial function, driving inflammatory states in neutrophils (30, 33). Notably, in the absence of platelets, neutrophils exhibit heightened surveillance activity, which could exacerbate chronic inflammation and fibrosis (34). This highlights the intricate interplay between platelets, neutrophils, and mitochondrial dynamics in immune regulation, as discussed further in the review.

## 4 Platelet roles in immune response and metabolic regulation

Platelets are recognized for their immune regulatory functions (54). Following injury, platelets facilitate vascular repair post-hemorrhage and recruit immune cells to the injury site, where infectious pathogens may enter (55). It is well-established that platelets interact with neutrophils through P-selectin, enhancing transendotelial migration and potentiating their immune functions (56). For example, during sepsis, platelets form clots in small vessels to prevent the hematogenous spread of infection, trap bacteria to facilitate their clearance, induce cellular differentiation, and present antigens, among other functions (57–59).

Beyond their mechanical roles in immune defense, platelets play a crucial role in inflammatory processes. Once recruited as single cells to the vascular wall, platelets act as pathfinders, guiding leukocytes out of the microvasculature to sites of inflammation (60). Several studies have shown that leukocytes rely on scanning activated platelets to pinpoint the exact locations for exiting circulation, highlighting the essential role of platelets in orchestrating leukocyte trafficking during inflammation (61, 62). While this review cannot cover the full range of platelet-mediated inflammatory mechanisms, others have thoroughly explored how platelets influence both inflammation and its resolution, as well as their impact on immune-mediated diseases (59, 63, 64). By discussing these selected mechanisms, we aim to illustrate the diverse ways in which platelets interact with other cells and processes, challenging the notion of platelets as simple or purely mechanical components of the immune system.

Platelets influence inflammation by secreting HMGB1, miR-15b-5p, and miR-378a-3p by means of exosomal release. These exosomal factors activate the Akt/mTOR autophagy pathway, leading to excessive release of NETs by neutrophils, which promotes inflammation and tissue damage (65). The role of platelets in driving NETs formation has been further demonstrated during dengue virus infections. Activated platelets numbers in infected patients are correlated positively with the presence of NETs, plasma leakage and worse prognosis (66). The proinflammatory functions of platelets appear to be regulated through multiple layers of control. For instance, platelet-derived factors have been shown to regulate IL-1β production by activating the inflammasome in various leukocyte populations (67). Additionally, activated platelets stimulate leukocytes to produce proinflammatory cytokines. In the case of SARS-CoV-2, the Spike protein induces the expression of P-selectin and CD40L on platelets, which bind to PSGL-1 and CD40 on monocytes, further promoting IL-1β production (68). This highlights the intricate role of platelets in amplifying inflammatory responses. Conversely, several key mechanisms have been identified that position platelets as gatekeepers of inflammation within the microvasculature, highlighting their cellular plasticity. On inflamed vasculature, platelets actively scan for fibrinogen in an Arp2/3-dependent manner to direct their spread and preserve vascular integrity, thereby preventing further inflammation (61). Furthermore, platelets opsonized with IgG can directly interact with circulating monocytes, inducing the production of anti-inflammatory IL-10 while simultaneously reducing the production of proinflammatory cytokines (69).

**Figure 1:**
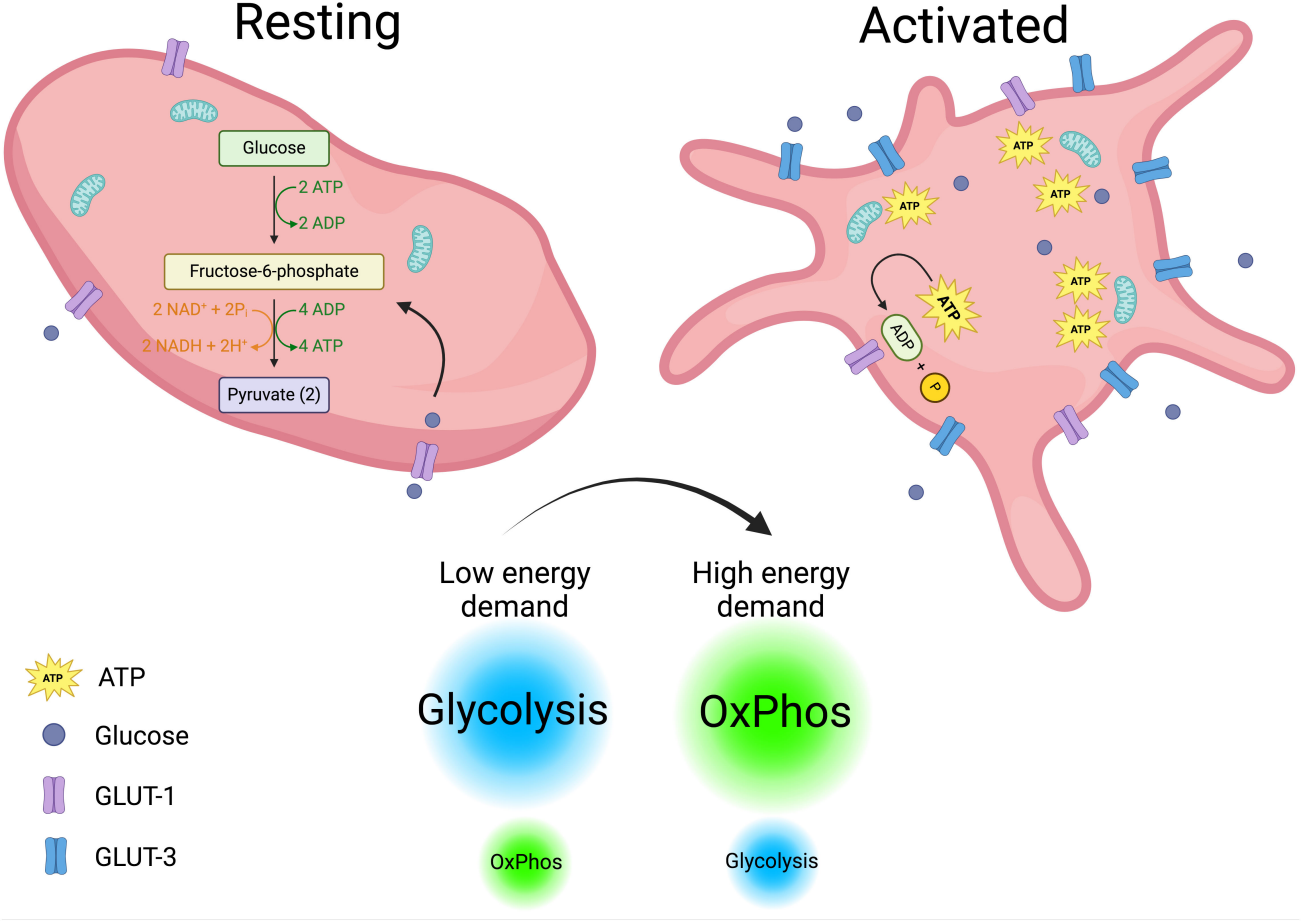
Platelets metabolism. Metabolic differences in resting and activated platelets are depicted. Resting platelets with a low energy demand rely mainly on glycolysis to fuel their functions. In contrast, activated platelets increase their energy demands and glucose intake, shifting from glycolysis to OxPhos (oxidative phosphorylation) to meet their energetic requirements. Created in https://BioRender.com.

The tight regulated immune functions that are just begun to envision for platelets, blurs the line that separate them from simplicity. To fulfill these diverse functions, platelets must be highly metabolically active (70, 71). Upon activation, their energy demands increase, prompting them to switch between metabolic pathways depending on the stimulus and available substrates (72). In a steady state, platelets primarily rely on glycolysis, with a lesser dependence on mitochondrial OxPhos (73). Upon activation, they increase glucose uptake through GLUT3, which is translocated from α-granule membranes to the plasma membrane. Notably, genetic deletion of GLUT3 impairs platelet activation and reduces degranulation, spreading, and clot retraction (74, 75). However, there is no clear consensus on which metabolic pathway primarily fuels platelet activation. Some studies report that aerobic glycolysis is the main pathway following platelet stimulation (70, 72, 76), while others suggest that oxidative metabolism is the key driver (77, 78). Corona-de-la-Peña et al. (2017) and Ravera et al. (2023) delve into these discrepancies, which may stem from variations in experimental approaches and readouts (71, 79). Additionally, platelets’ rapid and flexible switching between metabolic pathways contributes to the observed differences. Despite these variations, the importance of ATP as the ultimate energy source for platelet functions is undisputed. ATP is essential for processes such as maintaining calcium homeostasis, which activates key signaling pathways. Unsurprisingly, about 50% of mitochondrial activity in platelets is dedicated to ATP production (80). When OxPhos is pharmacologically inhibited, granule release and thrombus formation—two of the most energy-intensive platelet functions—are impaired (78, 79). Given the constantly changing conditions platelets face in circulation; their physiological functions should be viewed in this context. Rapidly shifting environmental factors may force platelets to rely on glucose through simultaneous use of glycolysis and OxPhos, occurring outside and inside the mitochondria, respectively. Supporting this idea, research indicates that platelets utilize glucose from two different sources with distinct metabolic fates (81). Metabolic differences in resting versus activated platelets are depicted in **Figure 1**.

## 5 Regulating neutrophil immunometabolism during inflammation

**Figure 2:**
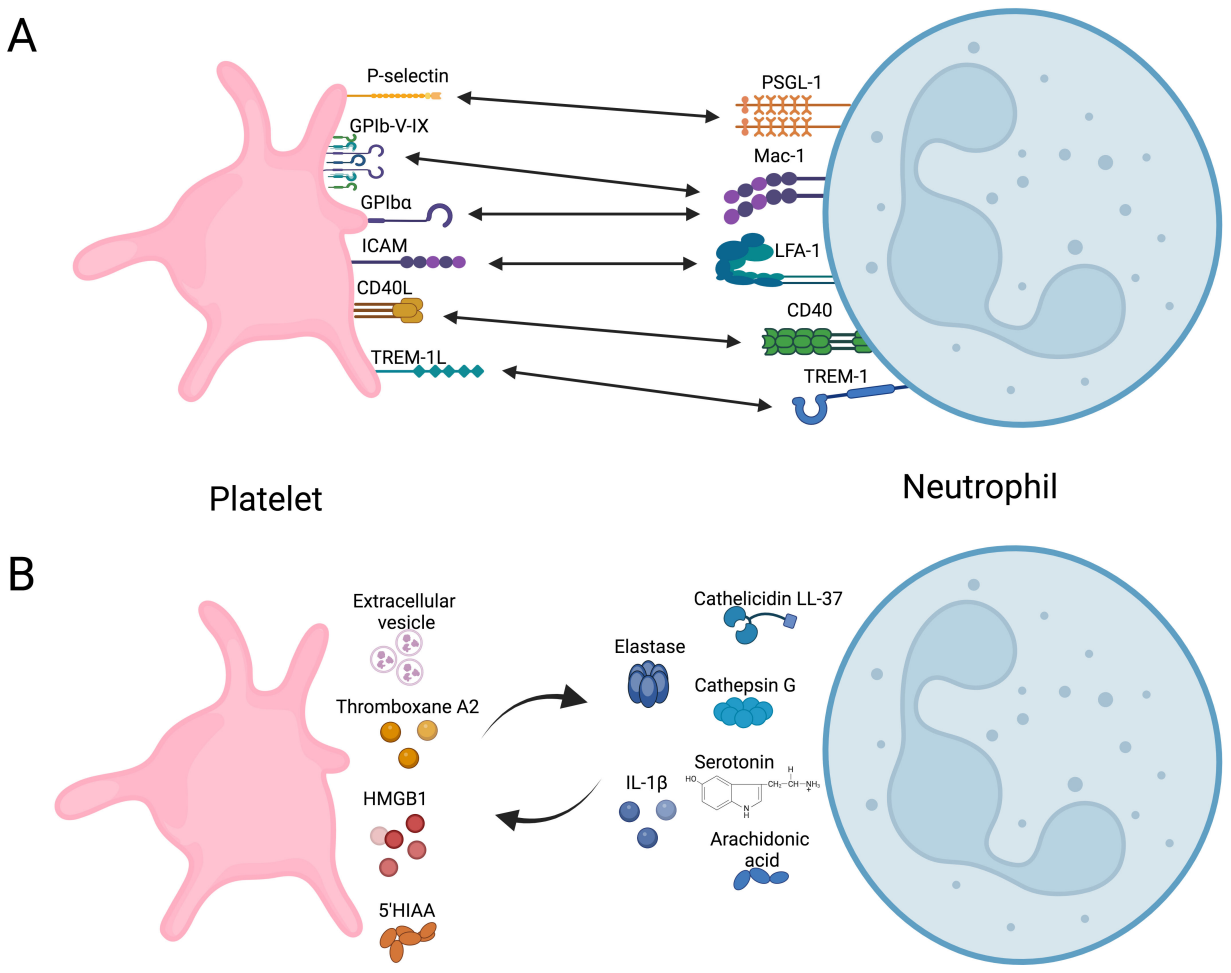
Overview of direct and indirect platelet-neutrophil interactions. **(A)** Receptors and ligand coupling that orchestrate bidirectional cellular activation. **(B)** Soluble molecules secreted by platelets that modulate neutrophils immune response and vice versa. HMGB1, High mobility group box 1; 5-HIAA, 5-Hydroxyindoleacetic acid. Created in https://BioRender.com.

Experimental evidence demonstrates that direct interaction between platelets and neutrophils, forming platelet-neutrophil aggregates (PNAs), enhances neutrophil function. This interaction bridges hemostasis and inflammation (55, 82–84), regulating neutrophil immune responses by modulating their metabolic pathways. Platelet-neutrophil receptor-ligand interactions are summarized in **Figure 2**, illustrating bidirectional activation mechanisms.

Platelet activation via ligands detected by TLR2, TLR4, or TLR9 induces the formation of PNAs (85, 86). This enhances the adhesion of neutrophils to vascular endothelium by increasing integrin affinity, promoting their migration into the tissue (84), and contributing to the resolution of infections caused by *Brucella abortus* (87), *Mycobacterium tuberculosis* (88), *Staphylococcus aureus*, and *Escherichia coli* (88, 89). These interactions trigger oxidative burst and the production and secretion of NETs. Platelet glycoprotein Ibα, P-selectin, and integrin αIIbβ3 are recognized by Mac1, PSGL-1, and SLC44A2 on neutrophils, respectively (56, 86, 90). However, triggering receptor expressed in myeloid (TREM)-like transcript-1 (TLT-1) and TREM-1 in platelets also play roles in this process (91, 92). TLT-1 serves as a specific marker for platelet activation, stored in α-granule and expressed on the surface after activation (93). TLT-1 regulates the transmigration of neutrophils during inflammatory events (91) by dissociating platelet-neutrophil aggregates before neutrophils transmigrate into the interstitial space. Additionally, TLT-1 promotes platelet adhesion to fibrinogen and contributes to fibrinogen accumulation (91). TREM-1 plays a significant role in neutrophil immunity (92). Interaction between PNAs and TREM-1 platelet-induced neutrophil activation, ROS production, and phagocytosis (92). During PNAs formation, platelets develop an activation phenotype characterized by increased expression of P-selectin and TLT-1. However, neutrophils regulate this activation phenotype on platelets to prevent procoagulant events (94).

Neutrophil immunometabolism is orchestrated by platelets to support membrane reorganization and cellular architecture, facilitating the production and secretion of NETs following PNAs formation. This metabolic shift involves a transition to ATP production via mitochondrial FAO (50, 51) rather than glycolysis, occurring in a NADPH-dependent manner (86). In obese mice, platelet dysfunction adversely affects the production and secretion of NETs. Restoring platelet functionality also reinstates the ability of neutrophils to produce and secrete NETs. In this murine model, the formation of PNAs modulates neutrophil influx, a process dependent on leptin, IL-33, and CXCR2 signaling driven by platelet secretory intermediates. Moreover, this process is inhibited by blocking P-selectin (50, 51). While intravital microscopy has proven valuable for examining neutrophil activity *in vivo* in mice (95), studying platelet-neutrophil interactions has required advanced tools, such as microfluidic assays (96), to better understand these complex mechanisms.

On the other hand, once PNAs are formed, a two-way communication begins. PAF activates phospholipase C in neutrophils, which hydrolyzes phosphoinositide substrate to produce inositol trisphosphate (IP_3_). IP_3_ binds to receptors on the endoplasmic reticulum, triggering the release of intracellular Ca²^+^. This increase in Ca²^+^ activates Ca²^+^-dependent phospholipase A_2_, which cleaves the sn-2 fatty acid from membrane phospholipids, releasing arachidonic acid (97). Neutrophils secrete arachidonic acid-rich extracellular vesicles (EVs), which are internalized by platelets in a Mac1-dependent manner. Subsequently, thromboxane A2 (TxA2) and other bioactive lipid mediators, including prostaglandins, and leukotrienes are synthesized by cyclooxygenase or lipoxygenase enzymes (97) and secreted, inducing endothelial expression of intercellular adhesion molecule-1 (ICAM-1) enhancing neutrophil transmigration (90), and oxidative burst through the increase of NADPH oxidase activity, and ROS generation (98). Moreover, histamine secreted by neutrophils regulates platelet activation by modulating Akt phosphorylation (99). Conversely, platelet EVs carrying transcription factors, nucleic acids, and containing mitochondria are internalized by activated neutrophils, thereby promoting inflammation (100). Furthermore, platelet recruitment of immune cells has been observed and is crucial for both initiating and resolving inflammation (34). Following neutrophil recruitment at the onset of inflammation, platelets recruit regulatory T cells (Tregs) during the resolution phase. Each mechanism relies on differential expression of P-selectin and activation by soluble CD40L. These aggregates of platelets and Tregs are vital for modulating their transcriptome and instructing Tregs to release anti-inflammatory mediators, such as IL-10 and transforming growth factor beta (TGF-β). As a result, macrophages undergo transcriptional reprogramming and polarization towards an anti-inflammatory phenotype, leading to effective resolution of inflammation mediated by TLT-1 expressed by platelets (34). Moreover, platelet-specific deletion of CLEC-2, but not GPVI, results in enhanced systemic inflammation and accelerated organ injury in two mouse sepsis models. This deficiency is linked to a reduction in podoplanin-expressing macrophages, despite elevated cytokine and chemokine levels in the infected peritoneum, suggesting that podoplanin activation mediates the anti-inflammatory effect of CLEC-2 on platelets regulating immune cell infiltration and inflammation during sepsis (101). Finally, the phosphorylation of dynamin-related protein 1 (Drp1) at Ser616, a key event in mitochondrial fission, regulates neutrophil polarization and chemotaxis. Platelet-derived factors may influence Drp1 phosphorylation, enhancing mitochondrial fragmentation in neutrophils. This process is linked to increased neutrophil activation and the formation of NETs, contributing to immune defense (30).

## 6 Platelet-derived metabolites and their influence on neutrophil responses

Metabolism is central to cellular function, and platelets are no exception. While the series of events during platelet activation seems to dictate which metabolic pathways are active, emerging evidence suggests that targeting specific metabolic pathways could modulate platelet functions (76, 78, 102, 103). Recent research has mapped the global metabolism of platelets following stimulation, revealing the intricate interplay between various metabolic pathways. In response to activation, 202 metabolites are upregulated, with lipids accounting for 50% of this increase (70). This section explores how platelet-derived metabolites impact neutrophil functions by modulating oxidative burst, survival, and metabolism (85, 86, 104).

**Figure 3:**
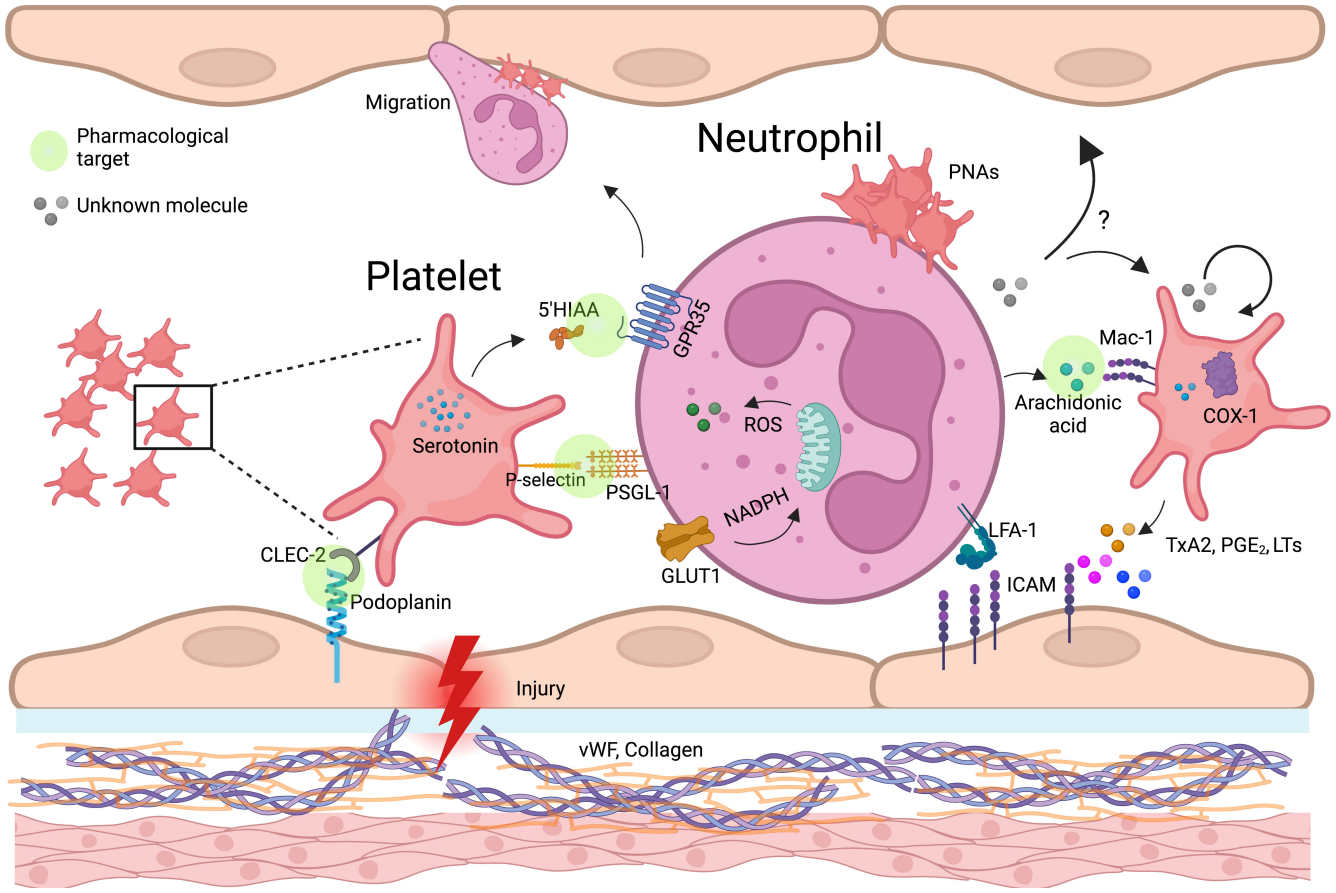
Platelet-neutrophil-endothelium crosstalk in vascular inflammation and immune regulation. Schematic representation of platelet-neutrophil-endothelial crosstalk in inflammation and vascular injury. Upon endothelial damage, platelets adhere to expose von Willebrand factor (vWF) and collagen, becoming activated and releasing key mediators such as serotonin, thromboxane A2 (TxA2), and arachidonic acid. Platelet P-selectin binds to neutrophil PSGL-1, facilitating platelet-neutrophil aggregates (PNAs). Neutrophils internalize platelet-derived arachidonic acid via Mac-1, leading to the production of lipid mediators (prostaglandins, leukotrienes) and reinforcing inflammatory responses. TxA2, produced by platelet COX-1, enhances endothelial ICAM-1 expression, promoting neutrophil adhesion and transmigration. Additionally, platelets transfer serotonin, which may modulate neutrophil oxidative burst through GPR35, and entry the mitochondria, potentially affecting neutrophil metabolism and immune function. Possible mechanisms, indicated by question marks (?), highlight critical knowledge gaps, such as the impact of neutrophil-derived metabolites in platelets (beyond arachidonic acid) and the endothelium. Green circle icons highlight the platelet-neutrophil-endothelium interaction points where pharmacological approaches can be developed to modulate the immune response during inflammatory diseases. Created in https://BioRender.com.

In the intestinal epithelium, low levels of IL-33 induce the release of serotonin by enterochromaffin cells, which is subsequently taken up by platelets. In conditions like chronic inflammatory bowel diseases (IBDs), where IL-33 concentrations are elevated, the heightened uptake of serotonin by platelets leads to increased clotting and neutrophil recruitment (105, 106). Platelets then metabolize serotonin into 5-HIAA, a key mediator in neutrophil recruitment through the GPR35 receptor (**Figure 3**). This mechanism has been validated in several murine inflammatory models. Additionally, 5-HIAA derived from platelets also aids in fungal clearance by promoting eosinophil recruitment (107, 108). Chemokines such as PF4, RANTES, CXCL4, and serotonin influence oxidative burst (104, 109), with PF4 specifically enhancing monocyte phagocytosis, prolonging ROS production, preventing apoptosis, and promoting cell differentiation (110). Another key mediator, HMGB1, supports neutrophil recruitment and activation by preserving mitochondrial potential, inducing autophagosome formation, and increasing NET production (49). Additionally, platelet-derived IDO1, a key enzyme in tryptophan metabolism, contributes to immune suppression by depleting tryptophan and generating kynurenine (111). Elevated IDO1 expression during *Plasmodium yoelii* infection alters plasma tryptophan and kynurenine levels, shaping immune responses of T cells, macrophages, and dendritic cells (112–114), while in severe malaria, excessive neutrophil activation exacerbates inflammation (115). Platelet-Derived Growth Factor (PDGF) induces mitochondrial fission in various cell types, including vascular smooth muscle cells (33). We hypothesize that PDGF may similarly promote mitochondrial fragmentation in neutrophils, a process linked to enhanced cellular responses such as oxidative burst and NET formation. Ahead of metabolic reprogramming, platelets can directly transfer functional mitochondria to neutrophils, influencing calcium mobilization, EVs release, and gene expression, thereby shaping immune responses (116).

**Figure 4:**
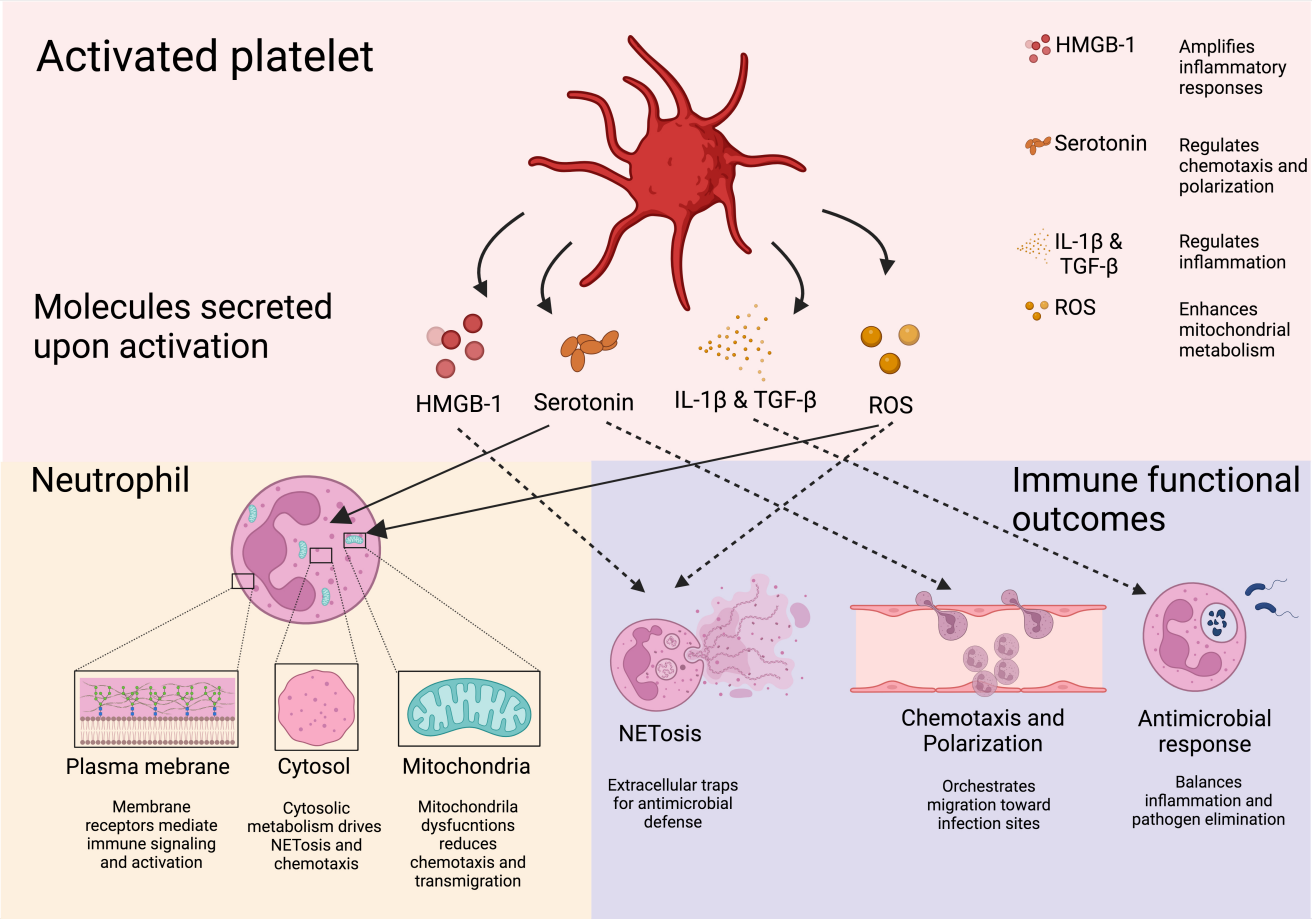
Soluble platelet-derive molecules regulate neutrophil immune response. Molecules derived from platelets and their effects on neutrophils are shown. Through their soluble mediators, platelets are able to induce neutrophil responses pivotal for the host defense. Downstream effects of platelet-derived molecules include NETosis, transmigration, and neutrophil recruitment. Created in https://BioRender.com.

Platelet-derived EVs modulate neutrophil function through multiple pathways, influencing both immune activation and metabolic reprogramming. EVs rich in soluble CD40L strongly enhance neutrophil oxidative burst via CD40-dependent activation of PI3K and NF-κB signaling (117–120). During dengue virus infection, platelets activated through CLEC2 release EVs that stimulate neutrophils via CLEC5A and TLR2, driving NET formation (121). Blocking these pathways significantly reduces the inflammatory response (121), suggesting a role for platelet-derived CD40L in regulating neutrophil immunometabolism by promoting oxidative burst and NET release (117, 118, 122). Additionally, EVs enriched in ceramides contribute to immune regulation by enhancing neutrophil migration, phagocytosis, and cytokine production while inhibiting the ETC, leading to ROS generation and apoptosis (123). Beyond neutrophils, platelet-derived lipid metabolites such as L-carnitine and acyl-carnitine influence immune cell metabolism. L-carnitine facilitates FAO and OXPHOS, increasing ROS production and activating FoxP3 signaling to enhance Treg suppressive function (123). Meanwhile, acyl-carnitine boosts mitochondrial ETC complex activity and OXPHOS in B cells by promoting H3K27 acetylation, thereby strengthening immunity (124) (**Figure 4**).

## 7 Impending challenges

Platelet transfusion is a lifesaving procedure implemented worldwide. Yet the clinical interest in platelets often remains limited to their role in hemostasis overlooking the broader physiological and pathological significance of these cells. However, cumulative evidence associates thrombocytopenia with increased mortality in critically ill patients (125, 126). While preclinical studies consistently highlight the regulatory roles of platelets during conditions such as sepsis, clinical studies have yet to confirm these findings conclusively (127). Notably, the immunomodulatory interactions between platelets and neutrophils offer a promising foundation for developing novel therapeutic strategies. Exploring the immunomodulatory potential of platelets on neutrophils for broader clinical applications is an emerging but challenging field (128). This attempt raises several interesting questions and presents key areas for future investigation. The first challenge involves elucidating the specific signaling pathways and molecular mechanisms underlying platelet-neutrophil interactions across various proinflammatory contexts. An interesting approach to tackle this matter would be the use of surfaceomics to define the molecule expression patterns on platelets and neutrophils from patients under specific scenarios, such as sepsis or dengue virus infection, where unregulated platelet-neutrophils interactions accounts for the progression of the disease (121, 129). The cell surface expression profile can be used as biomarker and would help to predict the outcome of a cell-cell interaction. The best characterized receptor-ligand interaction between platelets and neutrophils is P-selectin/PSGL-1. Notably, *in vitro* and clinical studies have demonstrated that human anti-P-selectin antibody can inhibit cell adhesion in a safety manner in individuals suffering from sickle cell disease (130, 131). These findings stablish a proof of principle on the importance of dissecting platelet-neutrophil interactions with a perspective of blocking their downstream effects to alleviate pathological conditions. The dynamic nature of platelet-neutrophil interactions, which may vary depending on disease stage, patient heterogeneity, and underlying metabolic conditions makes of this endeavor a hard to accomplish quest. In that context, to add a layer of complexity, is important to draw attention to the role of the endothelial cells (**Figure 3**). Critically, in vascular dysfunctions and systemic lupus erythematosus (SLE) (132), endothelial cells make important contributions to the onset and maintenance of the disease by interacting with platelet-neutrophil complexes. The second challenge is defining the roles of soluble platelet-derived molecules in neutrophil modulation. Recent studies revealed the metabolic complexity of platelets (70), highlighting a limited known number of metabolites that influence neutrophil immune responses. Given the heightened metabolic activity of activated platelets, it is reasonable to hypothesize that they produce a broader spectrum of metabolites capable of shaping the immune microenvironment beyond neutrophil modulation. Additionally, the reciprocal impact of neutrophil-derived metabolites on platelet functions and their contributions to immune regulation remain largely unexplored and warrant further investigation. From the short list of known platelet-derived metabolites, we highlighted the role of 5-HIAA on recruiting neutrophiles. 5-HIAA is a potent chemoattractan for all GPR35+ cells not just neutrophils which make it a promising candidate to exploit pharmacological immunomodulation during proinflammatory disorders. In recent years, significant advances in chemical compounds synthesis have allow the development of “peptidomimetics”, modified peptide sequences with improved biological properties (133, 134). The design of a peptide capable to block the action of 5-HIAA is appealing as treatment of viral respiratory infections including respiratory syncytial (RSV) and Influenza virus (135, 136), where, according to clinical studies, neutrophiles contribute to the lung damage during the disease. The third challenge emphasizes the need for comparative studies to analyze human and murine platelet-neutrophil interactions under similar conditions. These studies should aim to delineate species-specific differences in receptor functionality, metabolic pathways, and immune responses. Although, different in size and number, genetically human and mouse platelets share high levels of identity. Nevertheless, the differences exist. In silico analysis reveals that human and mouse secretomes are only 75% identical (137). Variations in receptor expression, metabolic dependencies, and signaling pathway sensitivities highlight the importance of cautious interpretation when translating murine model findings to humans. For example, variations in CLEC-2 expression or arachidonic acid metabolism between mice and humans could lead to differing immune outcomes. Validation using humanized models, organoids or primary human cells is imperative. While murine models remain indispensable for exploring complex *in vivo* interactions—particularly in inflammatory and thrombotic contexts—their genetic tractability must be balanced with efforts to ensure relevance to human biology. Comprehensively defining the impact of platelet-neutrophil interactions on immune responses holds significant relevance for human hyperinflammatory diseases such as sepsis and cardiovascular disorders. A clearer understanding of how platelet- and neutrophil-derived metabolites influence their crosstalk could have profound implications for basic physiology, immune regulation, and responses to infection.
